# Geospatial Overlap of Undernutrition and Tuberculosis in Ethiopia

**DOI:** 10.3390/ijerph20217000

**Published:** 2023-10-31

**Authors:** Fasil Wagnew, Kefyalew Addis Alene, Matthew Kelly, Darren Gray

**Affiliations:** 1 National Centre for Epidemiology and Population Health (NCEPH), College of Health and Medicine, The Australian National University, Canberra 2601, Australia; matthew.kelly@anu.edu.au; 2 College of Health Sciences, Debre Markos University, Debre Markos P.O. Box 269, Ethiopia; 3 Geospatial and Tuberculosis Research Team, Telethon Kids Institute, Nedlands 6009, Australia; kefyalew.alene@curtin.edu.au; 4 School of Population Health, Faculty of Health Sciences, Curtin University, Bentley 6102, Australia; 5 Population Health Program, QIMR Berghofer Medical Research Institute, Brisbane 4006, Australia; darren.gray@qimrberghofer.edu.au

**Keywords:** undernutrition, TB, geospatial overlap, Ethiopia

## Abstract

Undernutrition is a key driver of the global tuberculosis (TB) epidemic, yet there is limited understanding regarding the spatial overlap of both diseases. This study aimed to determine the geographical co-distribution and socio-climatic factors of undernutrition and TB in Ethiopia. Data on undernutrition were found from the Ethiopian Demographic and Health Survey (EDHS). Data on TB were obtained from the Ethiopia national TB prevalence survey. We applied a geostatistical model using a Bayesian framework to predict the prevalence of undernutrition and TB. Spatial overlap of undernutrition and TB prevalence was detected in the Afar and Somali regions. Population density was associated with the spatial distribution of TB [β: 0.008; 95% CrI: 0.001, 0.014], wasting [β: −0.017; 95% CrI: −0.032, −0.004], underweight [β: −0.02; 95% CrI: −0.031, −0.011], stunting [β: −0.012; 95% CrI: −0.017, −0.006], and adult undernutrition [β: −0.007; 95% CrI: −0.01, −0.005]. Distance to a health facility was associated with the spatial distribution of stunting [β: 0.269; 95% CrI: 0.08, 0.46] and adult undernutrition [β: 0.176; 95% CrI: 0.044, 0.308]. Healthcare access and demographic factors were associated with the spatial distribution of TB and undernutrition. Therefore, geographically targeted service integration may be more effective than nationwide service integration.

## 1. Introduction

Tuberculosis (TB) and undernutrition are major global health problems [1,2], with an estimated 768 million people suffering from undernutrition and 10.6 million cases of TB in 2021 [2,3]. The largest joint burden of undernutrition and TB exists in low-and middle-income countries (LMICs), with a significant concentration in sub-Saharan Africa (SSA) and Southeast Asia (SEA) [4]. At the global level and within specific countries, TB and undernutrition exhibit distinct geographic patterns of distribution. For instance, TB prevalence continues to be substantial in the SEA (44%) and the African regions (25%), and relatively low in the European region (2.5%) [5]. Similarly, the prevalence of undernutrition is high in Africa (20.4%) and in some parts of Asia (11.4%), and relatively low in Europe (2%) [6]. Given these geographical overlaps, gaining a deeper understanding of the connection between undernutrition and TB is crucial.

With limited resources and fragmented healthcare systems, Ethiopia also suffers significant challenges with both undernutrition and TB [7]. The occurrence of undernutrition among people with TB varies between 28.5 and 71.3% [8,9], which poses significant challenges to national programmes aimed at controlling and preventing TB [7].

The relationship between undernutrition and TB is bidirectional and complex. Undernutrition has been observed to be linked to an increased likelihood of developing TB [10,11], and most individuals with TB disease experience weight loss and nutritional deficiencies due to loss of appetite, nausea, and malabsorption [12]. A systematic review and meta-analysis suggested a 13.8% decrease in TB incidence for each unit increase in body mass index (BMI) [11]. The global TB report estimated that nearly 20% of new TB cases were attributable to undernutrition [4,13]. Particularly, undernutrition was responsible for one-third of TB cases in the 30 high-TB-burden countries [14], and over half of all TB cases in India [15]. Undernutrition is also associated with severe TB [16,17], including a high likelihood of adverse drug reactions, mortality, and treatment relapses [18]. This may indicate that the WHO End TB strategy’s goal by 2035, which seeks to reduce TB incidence by 95% and TB mortality by 90%, is less likely to be achieved without focusing on undernutrition.

The current end TB strategy paradigm mainly focuses on comprehensive public health interventions that address predictors for TB to complement the effectiveness of TB drugs [19]. While the diagnosis and treatment of TB remain essential in managing the disease, it is important to recognise the significance of addressing social determinants such as poverty, undernutrition, and food insecurity [20]. These factors shape the long-term environment and play a critical role in sustaining and improving the progress in reducing TB incidence. Addressing undernutrition and its impact on TB has the potential to provide opportunities not only for mitigating the TB epidemic but also hastening a cascade of improvement for public health generally and good progress toward the 2030 sustainable development goals (SDGs). For instance, improving an individual’s nutritional status through nutrition support enhances the effectiveness of TB drugs by boosting immunity and increasing drug absorption [18]. While the WHO issued guidelines on TB nutrition in 2013, there has been comparatively little emphasis on carrying out nutritional evaluations and applying tailored nutritional supports for individuals with TB [21]. The existing inadequacy of global progress in addressing TB, along with the increasing occurrence of multidrug-resistant TB (MDR-TB) and the numbers undernourished no longer declining, highlights the need for a new emphasis and approach. In addition, the health sector should engage in close collaboration with other sectors to ensure that the 2030 SDG targets related to TB can be achieved.

Identifying geographical overlap or high-risk areas for both TB and undernutrition is important to inform the implementation of cost-effective public health interventions [22,23,24,25,26]. The geographical distribution of TB and undernutrition is strongly influenced by shared demographic and socioeconomic factors [14]. TB is recognised as a disease associated with poverty and undernutrition, often arising from conditions of food scarcity and economic disadvantage. For instance, poverty, climatic change, and access to healthcare (and its quality) contribute to the double burden of TB and undernutrition [27].

However, the geospatial overlap of the two diseases at a local level is yet to be investigated in high-TB-burden and low-income countries. Therefore, recognising priority areas and the local spatial co-distribution of the two diseases in Ethiopia is essential for planning geographically targeted interventions for surveillance, treatment, and prevention. However, while some studies have investigated the spatial co-distributions of different diseases like TB, Human Immunodeficiency Virus (HIV), and malaria [24]; TB and HIV [28]; and diarrhoeal diseases and pneumonia [29]; at small area scales [30], no published reports show the geospatial overlap of TB and undernutrition.

Furthermore, although several small-scale studies in Ethiopia have described the relationship between undernutrition and TB at the individual level [31,32,33], the spatial co-distributions and ecological level drivers of undernutrition and TB at a lower administrative level are yet to be investigated. This study, therefore, set out to investigate the spatial co-distribution and socio-climatic factors of undernutrition and TB in Ethiopia.

## 2. Methods and Materials

### 2.1. Study Setting

**Figure 1:**
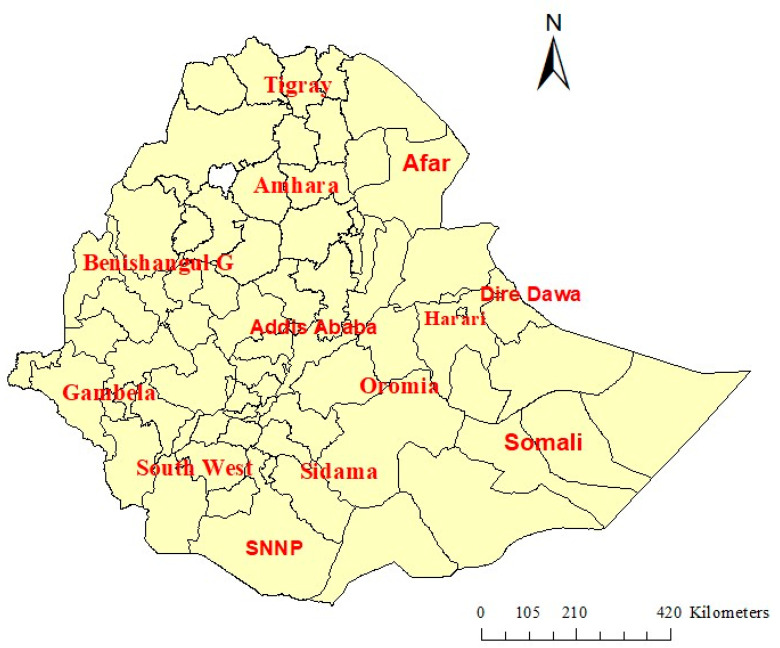
A zonal-level map of Ethiopia.

This study was carried out in Ethiopia, the second largest nation by population in Africa, which has approximately 115 million people and 13 administrative regions. The country has four administrative levels, which include regional states, zones, districts, and villages. Despite a significant 80% of the population living in rural areas, rural-to-urban migration is increasing. More than 85% of the population depends on agriculture and livestock husbandry. Ethiopia has diverse geographical characteristics, encompassing altitudes that extend from 125 m below sea level in the Afar region to a peak of 4620 m above sea level at Ras Dejen, and marked differences in healthcare coverage, disease burdens, and environmental conditions across the nation. Figure 1 illustrates a zonal map of Ethiopia (Figure 1).

The existing healthcare system in Ethiopia consists of a combination of private, governmental, and non-governmental sectors. Within the government healthcare service, there is a structured three-tier system consisting of primary-level care (comprising health posts, health centres, and primary hospitals), secondary-level care (including general hospitals), and tertiary-level care (consisting of specialised hospitals). TB diagnosis and treatment services are provided at all levels. The Ethiopian TB treatment guidelines include the integration of nutrition assessment, counselling, and support (NACS) to enhance the nutritional status of individuals [7].

### 2.2. Study Design

This study, conducted using an ecological analysis, was focused on assessing the geospatial overlap of the prevalence of both TB and undernutrition and the socio-climatic factors affecting these conditions. The investigation was carried out at a small geographical scale using a Bayesian analysis framework [34], which requires assigning appropriate prior distributions to all unknown parameters. Essentially, this means that parameter values are assumed to be governed by prior distributions and inference must be based on a posterior distribution that incorporates both likelihood and prior distributions. A full Bayesian analysis relies on examining the posterior distribution of the model parameters. This approach provides general information on parameter variability and between-parameter correlation. In our study, we estimated the Bayesian posterior marginal distribution using the Integrated Nested Laplace Approximation (INLA) and performed inferential analysis using R-INLA.

### 2.3. Data Sources and Outcomes of the Study

Our primary outcome measures were the prevalence of TB and undernutrition. Undernutrition comprised stunting, wasting, underweight, and adult undernutrition. For adults aged between 15 and 59 years, nutritional status was assessed by BMI. In addition, we included only women aged 15–49 years who were neither pregnant nor had given birth within the two months preceding the survey, and men aged 15–49 years.

For the primary outcomes and covariates, we used various data sources. Data for undernutrition were extracted from the Ethiopian Demographic and Health Survey (EDHS) collected in 2016. This survey was conducted using a sample that represented all regions of the country, distributed across a total of 645 clusters, of which 202 are urban and 443 are rural. The survey employed a two-stage stratified cluster sampling method, with the enumeration area and households designated as the primary and secondary sampling units, respectively. A comprehensive explanation of the sampling techniques for the EDHS can be found in another source [35].

Data on TB prevalence were sourced from the national TB prevalence survey conducted in Ethiopia, which is described in detail elsewhere [36]. Briefly, the TB prevalence survey was carried out in 2011 with a sample that provided nationwide representation. This community-based survey was performed using symptom assessment, chest X-ray examination, sputum smear microscopy, and sputum culture analysis among a total of 46,697 adults aged 15 years and above in 85 population clusters [37].

Potential socioeconomic and climatic factors were assembled from different sources. The selection of these variables was determined by the presence of nationally representative data offering a high level of spatial resolution and by previous studies demonstrating their association with both TB and undernutrition. Climatic variables, including mean annual temperature and mean annual precipitation, were sourced from the WorldClim website [38]. The Shuttle Radar Topography Mission database was used to obtain data for altitude [39]. Data regarding healthcare access (i.e., the time it takes to walk to the nearest healthcare facility in minutes) were obtained from the Malaria Atlas Project, and data for distance to a water body were obtained from the Global Lakes and Wetlands Database (GLWD) [40]. Population density, which quantifies the population count per grid cell, was derived from WorldPop [41]. Data for dietary diversity and adequate food availability were obtained from the EDHS. All of these data were retrieved with a 1 km^2^ spatial resolution. A polygon shape file for the Ethiopian administrative borders was extracted from the Global Administrative Areas database.

Data for the prevalence of underweight, stunting, wasting, and adult undernutrition were weighted using sampling weight to ensure an equal probability of selection in the EDHS data.

A geographical overlap area is defined as a geographical region exhibiting disease prevalence surpassing the upper quartile threshold of 75%. In the creation of a co-distribution map, the spatial predicted prevalence surface for each disease was overlaid in the GIS software. This method enables the identification of areas where the prevalence of two or more diseases coincides at its peak.

### 2.4. Operational Definitions

Table 1 contains the definitions for the primary outcomes, whereas Table S1 provides definitions for the included covariates.

### 2.5. Spatial Analysis

Spatially continuous predicted prevalence estimates across Ethiopia were generated for the outcomes of interest using Bayesian model-based geostatistics (MBG). Predicted prevalence models were constructed through binomial logistic regression analysis, incorporating space considered as a random effect and covariates as fixed effects.

Five different models were developed for the prevalence of wasting, underweight, stunting, adult undernutrition, and TB. Here, we describe how the model for the prevalence of wasting was developed. We followed a similar approach for the other models. We assumed that the number of children with wasting Y at each surveyed location *j* followed a binomial distribution:

*Y_j_* ∼ Binomial (*n_j_*, *p_j_*);

where n is the total number of participants and *p* is the predicted prevalence of wasting at location *j*. The prevalence at each location is then related to a linear predictor via a logit link function, whereby:

$$\mathrm{logit}\;(p_{j})=\alpha +\Sigma_{Ζ=1}^{Ζ}\beta_{Ζ}{Χ}_{Ζ,j}\zeta_{j}$$

In this context, α represents the intercept, *β* corresponds to the matrix of covariate coefficients, X stands for the design matrix of z covariates, and ζj represents the spatial random effects. These spatial random effects are characterised by a zero-mean Gaussian Markov random field with a Matérn covariance function [44].

Two parameters—ρ, which signifies the spatial scale representing the distance at which correlation becomes negligible, and σ, indicating the marginal standard deviation [45]—were used to determine the covariance function. Non-informative priors were specified for the intercepts. Additionally, for each covariate coefficient matrix, we set the mean to 0 and precision to 0.001. For the spatial random field parameters, we used a prior distribution, which was constructed by the INLA approach in R statistical software [45]. Bayesian inference based on INLA offers a computationally efficient alternative to Markov Chain Monte Carlo (MCMC), primarily designed to approximate MCMC estimates, especially in the context of latent Gaussian models. Sufficient values were run for each of the variables of interest, thereby ensuring a comprehensive characterisation of the posterior distributions. The model was used to predict the prevalence of wasting in unobserved areas across Ethiopia, using a prediction grid with a resolution of 1 km^2^. This involved summing the intercept, the spatial random effects, and the products of the coefficients and the observed values of the spatially varying fixed effects at each prediction location, followed by transforming the results from the logit scale to the prevalence scale. The results were imported into the GIS software to create predicted prevalence maps.

For model validation, we calculated the conditional predictive ordinates (CPO) and the probability integral transform (PIT) statistics. Models containing various covariate combinations were built and compared. The model with the lowest Watanabe–Akaike Applicable Information Criterion (WAIC) score was selected as the best-fitting model.

Data handling and analysis were performed using STATA/se version-17 and R version-4.2.0, and maps were created using ArcGIS version 3.4 software.

## 3. Results

**Figure 2:**
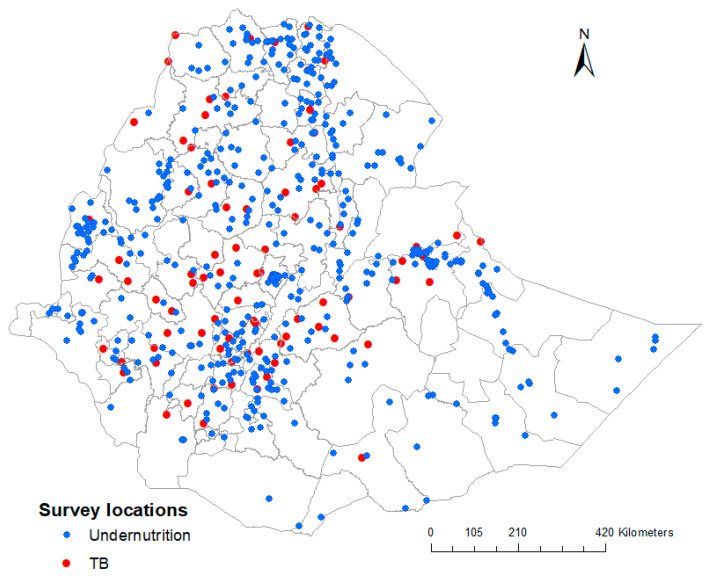
Geographical location of the surveys for undernutrition and TB in Ethiopia.

In total, we included 592 georeferenced clusters containing 9471 samples for stunting, 9596 samples for wasting, 9657 samples for underweight, and 20,345 samples for adult undernutrition; and 85 georeferenced clusters containing 46,697 samples for TB. The survey locations encompass all regions of the country for both undernutrition and TB, and are presented in Figure 2.

### 3.1. Factors at Socio-Ecological Level Associated with the Spatial Distribution of TB and Undernutrition Prevalence

The best-fitting models of socio-ecological-level factors associated with TB and all forms of undernutrition in Ethiopia are presented in Table 2. The model showed that population density had a positive association with TB [mean regression coefficient (β): 0.008; 95% CrI: 0.001, 0.014] and a negative association with wasting [β: −0.017; 95% CrI: −0.032, −0.004], underweight [β: −0.02; 95% CrI: −0.031, −0.011], stunting [β: −0.012; 95% CrI: −0.017, −0.006], and adult undernutrition [β: −0.007; 95% CrI: −0.01, −0.005].

We also found that distance to health facility had a positive association with stunting [β: 0.269; 95% CrI: 0.08, 0.46] and adult undernutrition [β: 0.176; 95% CrI: 0.044, 0.308] (Table 2). Other socio-ecological-level factors incorporated in the models; for instance, climatic variables, altitude, and proximity to a water body; did not show significant association with undernutrition or TB.

Comparison of models with different covariate combinations for spatial prediction of undernutrition and TB in Ethiopia are presented in the Supplementary Materials. The WAIC statistic was applied to validate model fitness, and the model that comprised all covariates was the best-fitting model for each disease (Tables S1 and S2).

### 3.2. Geospatial Distribution of Undernutrition and TB Prevalence in Ethiopia

**Figure 3:**
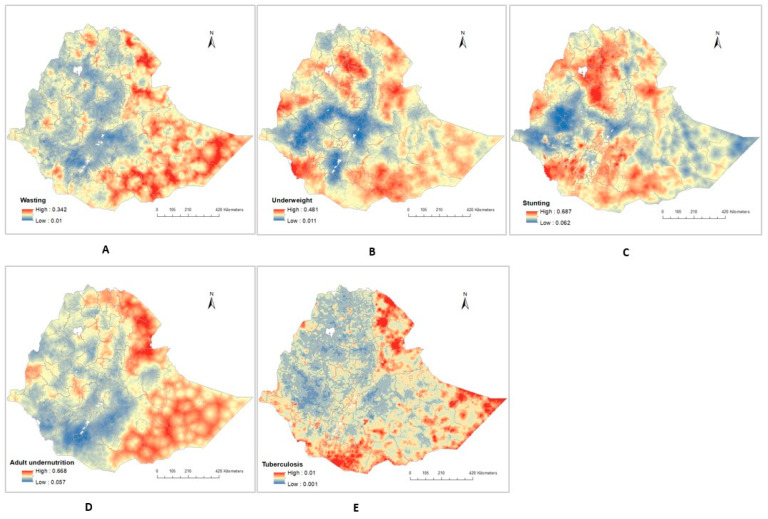
Predicted maps showing the spatial distribution in Ethiopia of: (**A**) Wasting, (**B**) Underweight, (**C**) Stunting, (**D**) Adult undernutrition, (**E**) Tuberculosis prevalence.

The prevalence of undernutrition (including wasting, underweight, and stunting) among children aged below five years old, and adult undernutrition at a high geographical resolution (1 × 1 km) is presented in Figure 3. The map showed substantial variations in the geographical patterns of wasting, underweight, stunting, adult undernutrition, and TB in Ethiopia at a pixel level.

The estimated prevalence of childhood wasting varied spatially, with the highest prevalence observed in the Somali, Dire Dawa, and Afar regions and the lowest prevalence observed in central Ethiopia. (Figure 3A). At a pixel level, the estimated prevalence of childhood wasting ranged from 1.0% to 34.2%.

The same trends appeared in the geographical patterns of adult undernutrition, with the highest prevalence at a pixel level (66.8%) observed in the Somali, Dire Dawa, and Afar regions and the lowest prevalence (5.7%) observed in central Ethiopia (Figure 3D).

There was also a substantial disparity in the prevalence of underweight, with the highest prevalence at a pixel level (48.1%) predicted in Somali, Afar, and certain areas of the Amhara region. The lowest prevalence at a pixel level (0.1%) was predicted in the Oromia and Gambela regions (Figure 3B). Tigray, Afar, Amhara, and some southern parts of the country were predicted to have a high prevalence of stunting; however, the Gambela region had a lower prevalence of stunting (Figure 3C).

We also found a large difference in the spatial distribution of estimated TB prevalence in Ethiopia. Across the country, estimated TB prevalence was generally higher in the peripheral regions (i.e., Afar and Somali regions) bordering Djibouti, Somalia, Eritrea, and Kenya; and it was lower in the central, northern, and western parts of the country (Figure 3E).

### 3.3. Spatial Overlap of Undernutrition and TB Prevalence

**Figure 4:**
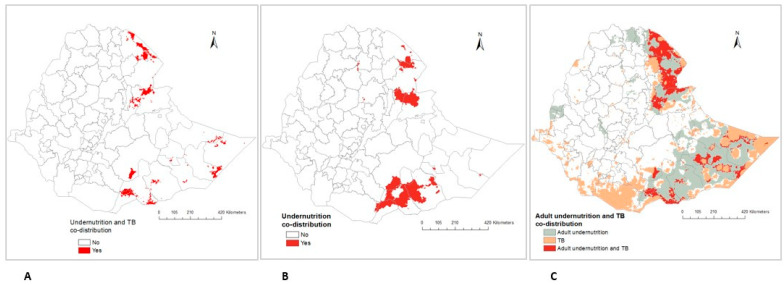
Predicted areas of geospatial overlap in Ethiopia: (**A**) All forms of undernutrition and TB, (**B**) All forms of undernutrition, (**C**) Adult undernutrition and TB.

Some parts of the country showed a geographical overlap in the predicted prevalence of undernutrition and TB (Figure 4). The burden of all forms of undernutrition and TB was highest in the specific areas within the Afar and Somali regions (Figure 4A). There was also a geographical overlap in the prevalence of all forms of undernutrition in some parts of the Somali and Afar regions (Figure 4B). In addition, the spatial co-distribution of high adult undernutrition and TB prevalence was detected in the Afar and Somali regions (Figure 4C). The geographical overlap of wasting and TB, underweight and TB, and stunting and TB are also described in the supplementary file (Figure S1).

## 4. Discussion

Our geospatial Bayesian models provide several insights into the epidemiology of TB and undernutrition in Ethiopia. The findings from this study demonstrate the predicted prevalence map for undernutrition and TB at local scales and identify their co-distributions, extending beyond previous research that has primarily focused on each aspect separately. Geographical co-distribution of TB and undernutrition was identified in some parts of the country. Substantial geographical variation was found in both undernutrition and TB prevalence in Ethiopia, which were determined by socioecological drivers. Our study highlights that population density was considered a consistent ecological-level risk factor associated with the spatial distribution of wasting, underweight, stunting, adult undernutrition, and TB. Distance to a health facility had a positive association with the spatial distribution of stunting and adult undernutrition.

In our study, we detected the highest prevalence of underweight in Somali, Afar, and some parts of the Amhara regions. The high predicted prevalence of wasting and adult undernutrition was also identified in Somali, Dire Dawa, and Afar regions, which is in line with previous studies [46,47]. These might be due to multifaceted factors. One possible contributing factor is access to safe drinking water, which is much lower in Afar and Somali regions. A previous study also demonstrated inadequate access to safe drinking water in these regions [48], which may in turn increase the burden of intestinal parasites, diarrhoeal diseases, and undernutrition. The highest inequality in access to minimum nutrition was found in the Somali and Afar regions, primarily because a majority of their food items are imported from other areas [49]. Inaccessibility of transportation further jeopardises the availability of imported nutritious foods.

The finding of our study regarding the geographical distribution of stunting is consistent with previous studies in Ethiopia, which consistently report a higher prevalence of stunting among children in the Tigray, Afar, and Amhara regions [50,51]. Studies conducted in Rwanda and Nigeria also showed a wide geographical variation of childhood stunting [52,53]. The highest stunting prevalence of the northern part of Ethiopia can possibly be attributed to political instability and environmental emergencies [54,55]. Geographically, the northern and eastern parts of Ethiopia have seen the most frequent drought episodes, locust plagues, and food crises [56], which might explain the higher prevalence of stunting [57]. This indicates that the national nutrition programme in Ethiopia must be comprehensive and focus on the specific geographical location with the highest prevalence of undernutrition.

We also identified a higher prevalence of TB in the peripheral regions that share international borders with Somalia, Kenya, Djibouti, and Eritrea, while a lower TB prevalence was detected in the central, northern, and western parts of the country. This finding aligns with other studies that detected hotspot areas in the country’s outermost areas [24,58]. There is a considerable risk of TB transmission in the bordering areas due to inadequate diagnosis and management [59]. It is noteworthy that the identification of a high prevalence of TB in the border areas indicates the existence of shared common TB risk factors and highlights the value of identifying the dynamics of TB transmission across districts [60,61].

In this study, a substantial geographical co-distribution of undernutrition and TB prevalence was detected in the Afar and Somali regions. These two regions, which are predominantly pastoral, have poor infrastructure and inequality of access to health services. The national survey report also showed that, in terms of health facilities and infrastructure coverage, the Somali regional state performed poorly [62]. As has been noted, the majority of the Somali and Afar regional areas are facing a recurrence of drought and decreased rainfall distributions [55,63], where crop production is threatened, resulting in food insecurity [64,65]. This is particularly common in SSA countries such as Ethiopia, where crop production primarily depends on rain-fed agriculture [66]. This community also has limited access to social services, particularly in the areas of education, healthcare, and water supplies [48,67]. In addition, it is plausible that individuals with lower income levels may face challenges in acquiring nutritionally balanced diets and could be more likely to reside in slum areas with overcrowded and unsanitary living conditions. Furthermore, limited access to healthcare services in such areas can potentially heighten their vulnerability to both malnutrition and TB. Therefore, our findings indicate that the national and regional healthcare planner for TB and undernutrition should focus on the northeastern and southeastern parts of the country. Urgent measures are required to address the sustainable enhancement of water irrigation for crop cultivation and expand the range of agricultural output as a means to reduce the burden of undernutrition. Moreover, our analysis suggests the need for strengthening the TB-nutrition programmes in the country, particularly in the Afar and Somali regions. An integrated intervention approach should encompass enhanced early screening and diagnosis of the two diseases, providing nutritional and social support services; including expanding healthcare access, financial incentives, and access to education; to mitigate the socioeconomic factors that increase vulnerability to both diseases. This involves collaboration between healthcare professionals, nutritionists, and social workers. Such interventions can improve health outcomes, reduce healthcare costs, and enhance the overall well-being of affected individuals.

In the current study, we identified demographic factors that influence the geographical distribution of TB and undernutrition. Population density had a positive association with TB prevalence and a negative association with underweight, wasting, stunting, and adult undernutrition prevalence. Previous studies reported that an area with a high population density (urban population) was shown to have a high prevalence of TB, as a crowded area is a risk factor for transmission [24]. Conversely, we found that undernutrition was higher in rural areas than in urban areas, findings consistent with other studies conducted in Indonesia [68], Bangladesh [69], and Tanzania [70]. Studies on urban–rural differences in undernutrition suggest that those living in cities have good nutritional status when compared to those living in rural areas [71,72]. This discrepancy can be attributed to factors such as improved access to food, better housing conditions, greater employment opportunities for parents, and the availability of essential amenities like electricity, clean water, and sanitation facilities [71]. Additionally, cultural influences, limited awareness of dietary diversity, and inadequate access to healthcare services may contribute to these observed variations [67,73,74]. There is a clear indication that programmatic interventions, such as the expansion and acceleration of efforts to address undernutrition, should be prioritised and extended specifically in rural areas. In particular, nutrition activities across sectors, especially in the current context of TB and nutrition, will need to be supported and equitably expanded. Implementing integrated interventions in rural areas would not only benefit the targeted communities but also prevent the potential transmission of TB to urban areas. These integrated interventions usually encompass the strengthening of healthcare systems, improvement in education access, and other opportunities in rural settings, leading to enhanced healthcare accessibility, early TB identification, and treatment. They can contribute to a reduction in rural-to-urban migration, thereby diminishing the transmission of TB from rural to urban populations. In light of the growing trend of rural-to-urban migration in Ethiopia, it is reasonable that migrants may bring their awareness of infectious diseases such as TB and HIV and good nutritional status, consequently reducing the risks of latent TB reactivation and transmission in urban settings.

Furthermore, this study revealed a positive association between distance to a health facility and stunting and adult undernutrition. Communities who live far from health institutions may have inadequate knowledge about dietary diversity and infection prevention measures, and a lack of awareness that affects the health-seeking behaviour of individuals. Previous studies confirmed inequalities in access to health facilities and the distribution of healthcare providers in rural areas [75,76,77]. Although nutritional counselling and TB diagnostic testing are available in healthcare facilities in Ethiopia, coverage is poor and unevenly distributed across the country [73,78]. Moreover, communities far from health facilities may be more likely to choose traditional medicine [79], which may delay diagnosis. In contrast, accessible healthcare services enable individuals to receive regular monitoring, follow-up visits, medication adherence support, and nutritional counselling. Therefore, our study indicates that identifying geographic areas at higher risk of undernutrition and TB, and putting supportive strategies in place; such as expanding social media platforms, and health service coverage with equally distributing healthcare workers in under-served areas; will have substantial contributions to mitigating the burden of undernutrition and TB in Ethiopia.

### Limitations of the Study

Despite an advanced geospatial model being fitted to present the first results describing the co-distribution of TB and undernutrition prevalence in Ethiopia at local scales, this study has some limitations. We attempted to investigate the geospatial overlap of TB and undernutrition using data from the 2016 EDHS and the 2011 TB surveillance data. Nevertheless, there was a discrepancy in the time frames of the data sources used, due to the fact that conducting nationwide TB prevalence surveys on a regular basis needs high resourcing and has logistical complexities. As a result, many recent studies utilised these survey data to provide nationally representative estimates of TB prevalence, especially for applying spatial analysis, as this model needs a large sample size and statistical power to minimise uncertainty. It is therefore important for future studies to acknowledge and address this limitation by utilising the most recently available national TB surveillance data, ideally from the same year as the EDHS data sources, when they become accessible.

Certain important covariates, such as economic factors, were not incorporated in the model due to the fact that these data are not available. We were also unable to predict the prevalence of TB among children in the country due to the absence of comprehensive, nationally representative data. Additionally, it is important to acknowledge that the location information related to the data used for this study may contain errors. For confidentiality reasons, many surveys that collect Global Positioning System (GPS) coordinates employ a technique of random displacement on those coordinates before releasing the data for secondary analysis. To illustrate, GPS coordinates for the DHS experience displacement of up to 2 km for urban clusters, up to 5 km for the majority of rural clusters, and up to 10 km in approximately 1% of randomly selected rural clusters [37]. Previous research has indicated that this displacement can have a slight detrimental effect on the model’s accuracy. Nonetheless, researchers have determined that reasonably accurate mapping can still be achieved at a 5 × 5 km resolution even when GPS displacement is applied [80].

## 5. Conclusions

There were substantial variations in the prevalence of TB and all forms of undernutrition (i.e., underweight, wasting, stunting, and adult undernutrition) in Ethiopia at local levels, which were significantly associated with distance to a health facility and population density. Spatial overlap of undernutrition and TB prevalence was identified in certain regions of the country, notably in the Afar and Somali regions.

This study provides relevant evidence that can inform decision-making and service delivery strategies aimed at enhancing the prevention and management of TB. More attention should be placed on expanding healthcare access and community health extension programmes (HEPs), particularly in areas that are remote and rural. In addition, this study highlights the need for designing integrated and targeted public health strategies addressing marginalised populations. Investigating the spatiotemporal patterns of TB and undernutrition, considering both space and time, would also be ideal in the future.

## Figures and Tables

**Table 1:** Operational definitions.

Variable	Definitions
Stunting	Nutritional status is determined by assessing the height-for-age Z-score, which falls below minus two standard deviations (-2SD) from the WHO Growth Standards median [42].
Underweight	Nutritional status is determined by assessing the weight-for-age Z-score, which falls below minus two standard deviations (-2SD) from the WHO Growth Standards median [42].
Wasting	Nutritional status is determined by assessing the weight for height Z-score, which falls below minus two standard deviations (-2SD) from the WHO Growth Standards median [42].
Adult undernutrition	Nutritional status based on BMI value, which is below 18.5 kg/m^2^ [25,36,43].

**Table 2:** Regression coefficient mean and 95% CrIs of socio-climatic factors considered in a Bayesian spatial model for wasting, underweight, stunting, adult undernutrition, and TB prevalence in Ethiopia.

Covariates	Wasting	Underweight	Stunting	Adult Undernutrition	TB
	Regression Coefficient Mean 95% CrIs	Regression Coefficient Mean 95% CrIs	Regression Coefficient Mean 95% CrIs	Regression Coefficient Mean 95% CrIs	Regression Coefficient Mean 95% CrIs
Temperature (°C)	−0.425(−1.012, 0.136)	−0.233(−0.706, 0.234)	0.213(−0.239, 0.667)	−0.128(−0.464, 0.206)	−0.492(−1.41, 0.424)
Precipitation (millimetre)	−0.136(−0.331, 0.063)	0.035(−0.17, 0.277)	0.149(−0.078, 0.403)	0.012(−0.161, 0.201)	−0.206(−0.551, 0.071)
Altitude (metre)	−0.51(−1.113, 0.065)	−0.268(−0.763, 0.216)	0.346(−0.126, 0.815)	−0.244(−0.596, 0.104)	−0.667(−1.65, 0.323)
Population density (people per grid cell)	**−0** **.017(−0** **.032, −0** **.004)**	**−0** **.02 (−0** **.031, −0** **.011)**	**−0** **.012(−0** **.017, −0** **.006)**	**−0** **.007(−0** **.01, −0** **.005)**	**0** **.008(0** **.001, 0** **.014)**
Distance to water body (kilometre)	−0.03(−0.11, 0.05)	−0.039 (−0.096, 0.018)	−0.016(−0.068, 0.036)	−0.018(−0.053, 0.015)	0.076(−0.104, 0.252)
Distance to a health facility (minutes)	0.071(−0.196, 0.332)	0.176(−0.026, 0.379)	**0** **.269(0** **.08, 0** **.46)**	**0** **.176(0** **.044, 0** **.308)**	−0.276(−0.866, 0.29)
Enough food availability	−0.054(−0.169, 0.06)	−0.076(−0.169, 0.014)	−0.037(−0.126, 0.05)	−0.013(−0.078, 0.05)	0.148(−0.028, 0.317)
High Dietary diversity 6–23 months	−0.023(−0.183, 0.137)	0.136(−0.008, 0.291)	0.12(−0.019, 0.264)	-	-
Intercept	−2.02(−2.29, −1.776)	−1.05(−1.325, −0.77)	−0.502(−0.83, −0.16)	−0.734(−1.003, −0.47)	−5.64(−6.52, −4.9)

CrIs: credible intervals. The numbers in bold indicate ‘statistically significant’ results within a Bayesian framework. Dietary diversity data were not available for adult undernutrition and TB.

## Data Availability

All data produced or scrutinised throughout this study have been incorporated within this article [and its supplementary information files].

## Supplementary Materials

The following supporting information can be downloaded at: https://www.mdpi.com/article/10.3390/ijerph20217000/s1, Table S1: Sources of data and definitions for covariates. Table S2: WAIC values related to various models. Figure S1: Predicted areas of geospatial overlap of stunting and TB, underweight and TB, and wasting and TB.

## Abbreviations

BMI	Body Mass Index
EDHS	Ethiopian Demographic and Health Survey
GPS	Global Positioning System
HIV	Human Immunodeficiency Virus
MCMC	Markov Chain Monte Carlo (MCMC)
LMICs	Low- and Middle-Income countries
SSA	Sub-Saharan Africa
TB	Tuberculosis
WAIC	Watanabe–Akaike Applicable Information Criterion
WHO	World Health Organisation
