# Supplementary material for: Geospatial Overlap of Undernutrition and Tuberculosis in Ethiopia
Source: Int J Environ Res Public Health. 2023 Oct 31;20(21):7000. doi: 10.3390/ijerph20217000 (PMC10647613; doi:10.3390/ijerph20217000)
Supplement: Supplementary file 1 [file ijerph-20-07000-s001.zip › Table S1.pdf]

Table S1: Data sources and definitions of covariates.

Covariates	Data sources	Definitions
**Population density**	WorldPop	Number of people per square kilometre (grid)
**Temperature**	WorldClime	Annual mean environmental air temperature (°C)
**Precipitation**	WorldClime	Annual mean rainfall (mm)
**Altitude**	Shuttle Radar Topography Mission (SRTM)	Elevation of the earth land surface in kilometre
**Distance to a water body**	Global Lakes and Wetlands Database (GLWD)	Distance to permanent and semi-permanent water based on presence of lakes, wetlands, rivers and streams, and accounting for slope and precipitation
**Access to healthcare facilities**	Malaria Atlas Project (MAP)	Walking travel times in minutes to the nearest hospital or clinic
**Available of enough food**	Ethiopian Demographic Health Survey Datasets (EDHS)	The availability of sufficient quantities of food of appropriate quality, supplied through domestic production or imports
**Dietary diversity**	Ethiopian Demographic Health Survey Datasets (EDHS	A qualitative measure of food consumption that reflects household access to a variety of foods
