# Supplementary material for: Geospatial Overlap of Undernutrition and Tuberculosis in Ethiopia
Source: Int J Environ Res Public Health. 2023 Oct 31;20(21):7000. doi: 10.3390/ijerph20217000 (PMC10647613; doi:10.3390/ijerph20217000)
Supplement: Supplementary file 1 [file ijerph-20-07000-s001.zip › Table S2.pdf]

Table S2: Watanabe-Akaike information criterion (WAIC) values corresponding to different model specifications.

Model specifications	Wasting	Underweight	Stunting	Adult undernutrition	TB
Temperature	1432.573	2005.163	2130.001	2848.997	311.7481
Temperature + Precipitation	1430.553	2004.048	2123.506	2852.763	313.6291
Temperature + Precipitation + Altitude	1424.404	2005.724	2124.449	2855.33	314.0912
Temperature + Precipitation + Altitude + Distance to water body	1424.125	2007.913	2121.687	2859.084	313.147
Temperature + Precipitation + Altitude + Distance to water body + Population density	1418.422	1999.163	2104.37	2816.152	315.414
Temperature + Precipitation + Altitude + Distance to water body + Population density + Distance to health facilities	1420.159	1998.856	2107.225	2808.21	317.2598
Temperature + Precipitation + Altitude + Distance to water body + Population density + Distance to health facilities + Enough food available	1419.063	1999.403	2102.997	2811.138	318.2083
Temperature + Precipitation + Altitude + Distance to water body + Population density + Distance to health facilities + Enough food available + Dietary diversity	418.06	1994.23	2100.11	-	-
