# Supplementary figures and images for: Geospatial Overlap of Undernutrition and Tuberculosis in Ethiopia
Source: Int J Environ Res Public Health. 2023 Oct 31;20(21):7000. doi: 10.3390/ijerph20217000 (PMC10647613; doi:10.3390/ijerph20217000)

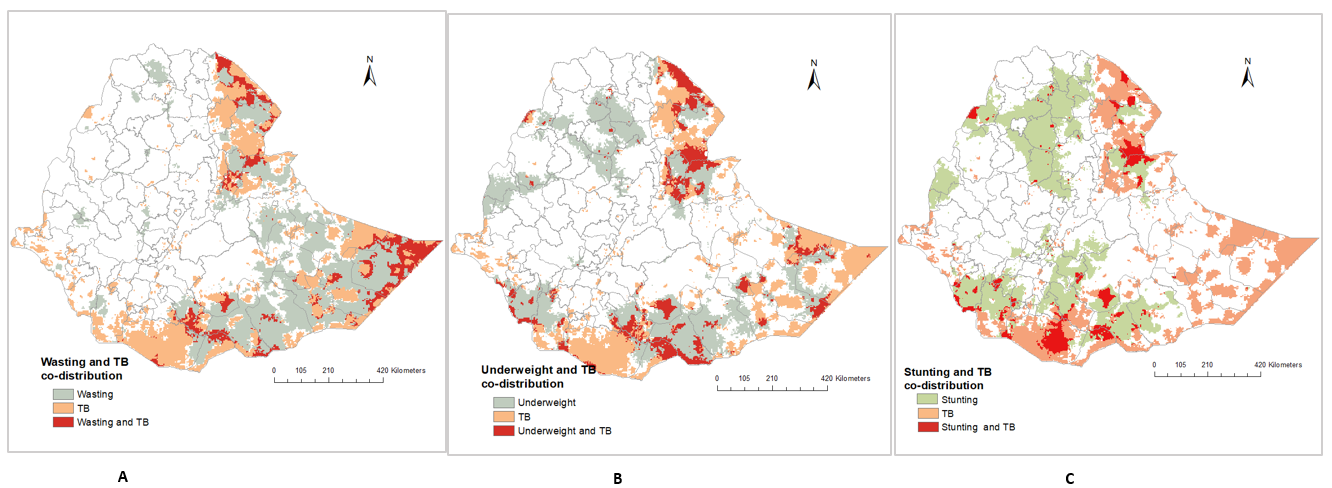

Supplement: Supplementary file 1 [file ijerph-20-07000-s001.zip › Figure_S1.png]
